# In a Coal Mine with Miners.—II

**Published:** 1889-03-16

**Authors:** 


### IN A COAL MINE WITH MINERS.—*(Concluded from p. 361.)*

BY A NEW HAND.

II.

MEN, WORK, AND WAGES.

THE miners proper may be divided into stall-men, or "butties," with their assistants, and "rippers," who have three or four men under them. The stall-men, or "butties," direct and assist in the work of getting the coal from the face of the mine. This work is, at times, very laborious, as they have not only to pick the coal out, and to place it in the trucks, but to build up the dirt behind them as they work, to form the "goaf," or "gob," leaving the gates on either side, whilst the roof is supported by timber planking. The butties are paid by the ton of coals raised, the average price in Derbyshire being about 2s. 6d. a-ton. The "rippers" have three or four men under them, and it is their duty to "rip" or cut the gates, and to rip and pack the main roads, all of which work is done during the night. They are paid by the yard, the price in Derbyshire being about 6s., which sum includes everything, as the "ripper" has to pay all outgoings in the shape of wages to his assistants, and so forth. It is estimated that the wages which it is possible for these men to make in good times, by continuous work, varies from 35s. to £3 a-week. It may be added that the height of the road is from six to eight feet, and of the gates from three feet to four feet six inches. The roads are built up much more carefully than the gates, being arched with bricks as they approach the main entrance to the bottom of the shaft. The gates vary from four to eight feet at the start, but they soon settle down to about three feet, when it is necessary to cut out another foot or so to enable the work of the mine to proceed without difficulty.

DANGERS TO BE AVOIDED.

These are always, present, and are very real as a visitor soon finds out. It can be imagined that neither the roads nor the gates are wider than it is absolutely necessary to make them, and hence the space left for such superfluous matters as the body proper of a casual visitor, when a train of trucks has to be passed, is not unduly large. The regulations provide that refuges—that is, recesses—shall be made at every few yards, but this fact has to be known, and their locality ascertained, before they inspire confidence. Even then great danger arises, at times, to the regular workers, and especially after a strike. This is due to the freshness of the horses and ponies, who, having been maintained without work for days or weeks, as the case may be, frequently become unmanageable, and, in this condition, take to running away. Imagine the feelings of a stranger in a narrow gate, some five feet wide by four feet high, surrounded by darkness, when he becomes aware that a runaway horse, with a train of trucks behind him, is coming towards him at a break-neck pace. The mere mention of such a fact by our engineer guide, as we proceed with difficulty along one of these narrow passages, groping our way in the dark, is well nigh sufficient to prevent further progress, and to reduce the nerves of the visitor to a condition which makes him start and jump whenever he hears a train of trucks approaching. Another danger, and a very real one, is that which arises from falling roofs. The goodly apple rotten at the core—the villain with the smiling face—is met with in mines as well as on the earth's surface. In the former he takes the shape of a piece of rock, with a highly polished surface, which looks as solid and as safe as adamant. Despite this outward appearance of stability, these smiling villains fall upon the unwary without notice, and when least expected. Lastly, there is that most appalling danger, choke-damp, or gas, which, in some seams of coal, becomes generated at a rate which makes it highly dangerous to work them without the utmost precautions being taken, and which renders carelessness fatal to the workers. A proper system of ventilation, which must be constantly regulated and inspected, alone renders it possible to work coal of this description. In Derbyshire, as we have said, this kind of coal is seldom or never met with, and, although the earnings of the miners are less, owing to the undue proportion of dirt which is mingled with the coal, it is gratefully remembered by the men that here the dangers arising from the presence of choke-damp or gas are reduced to a minimum.

MINER'S IDIOSYNCRACIES AND CREEDS.

On leaving the deputy's office to enter the mine proper, we noticed two able-bodied miners lying, with their assistants, half-asleep, a little way to the left. The engineer inquired what they wanted, and proceeded to explain that they were new hands, who had entered the mine, as workers, for the first time that morning. They replied, "Too much dirt; we ain't a going to work in such conditions for nobody." These were Yorkshiremen, who preferred the dangers of the full seam of coal to the comparative safety, but increased labour of the Derbyshire dirt and coal combination. Asked if there were many such cases, the engineer replied that in all probability, as the men we saw would have to remain the whole of the eight hours of the working day in the pit doing nothing, no one being allowed to leave it until work ceased, they would think the whole matter out, return to work the next morning at the very same place which they now complained of, and continue to labour in the mine for several years without a murmur. Whitsuntide, and other influences of a like nature, affect the miner's temper and views of life as they do those of many other people. Arrived at the face of the mine, we came to a "buttie" called Tom, who had worked in various pits for thirty years past. He was an active and intelligent man, of sporting propensities, who took a cheerful view of his work and of things generally. He had backed Borneo for the Manchester Cup, and now lamented that he had "hedged" £5, and so had won only £7 instead of £12, which he would otherwise have received. It was three days before the Derby, and Tom strongly advised us to back Melton, as he had done, and assured us he must win, and nothing could stop him. Melton, of course, did pass the post first. May this be taken as evidence that coal-mining tends to contemplation, and so to successful prophecy?

A WORD FOR THE MINERS.

Everyone has heard of the reckless expenditure of miners, and of their disregard for the proprieties of life. How the baby is turned out of the cradle to make way for the bull pup; how ducks and green peas are eaten at a season whenthey can only be secured by Dives at a ruinous cost; and how man and dog fights afford an unfailing attraction to a mining population. Thinking over these things as we left the coal pit, we saw a group of small houses within a stone's throw of the furnaces, where many of the workers and their families reside. Miles away from any place of amusement, closely adjacent to the dirt and smoke, and the bleak aspect which mining operations produce, the toilers and moilers who reside in these houses seem to possess no means of relaxation of a rational character. Our thoughts carried us to Pullman Town, west of Chicago, with its theatre, concert-room, gymnasium, cricket and rowing clubs, *et hoc genus omne*, where 15,000 workers reside with their families, and live in happiness, contentment, and peace, without the restraining influence of even a single policeman. It is true that, of late years, it has become increasingly the fashion to promote organisations for providing amusement and relaxation for the people. A good deal has recently been said of the responsibilities of noblemen and landowners. Might not, at least as much be said concerning the responsibilities and duties of colliery proprietors, and large employers of labour? Capital conduces to comfort in the possessor, but it entails the responsibility upon its owner of securing for the bees who gather the honey, a reasonable proportion of the relaxations and enjoyments of life. The miners' village should not be left devoid of all means of amusement and relaxation for its inhabitants, except those derived from the public-house. Having seen the miner at work, having realized a little of the hardships of his toil, and the absence of a kindly hand to provide him with rational relaxation, the writer's sympathies have been excited, as he hopes those of the reader may be also, and that in the result a radical change in the direction indicated may speedily be effected.

